# History of Developing Acute Promyelocytic Leukemia Treatment and Role of Promyelocytic Leukemia Bodies

**DOI:** 10.3390/cancers16071351

**Published:** 2024-03-29

**Authors:** Pierre Bercier, Hugues de Thé

**Affiliations:** 1 Center for Interdisciplinary Research in Biology (CIRB), Collège de France, CNRS, INSERM, Université PSL, 75231 Paris, France; hugues.dethe@inserm.fr; 2 GenCellDis, Inserm U944, CNRS UMR7212, Université Paris Cité, 75010 Paris, France; 3 Hematology Laboratory, Hôpital St Louis, AP/HP, 75010 Paris, France

**Keywords:** acute promyelocytic leukemia, PML, nuclear bodies, arsenic, all-trans retinoic acid, targeted therapy

## Abstract

**Simple Summary:**

Acute Promyelocytic Leukemia (APL) was the most aggressive form of leukemia, historically associated with massive and rapid mortality. However, empirical clinical advancements and in-depth mechanistic exploration have now transformed APL into the most curable form of leukemia, culminating with the ATRA/ATO combination, which cures up to 98% APL patients. This review recapitulates a three-decade journey which led to the development of this groundbreaking treatment, through several paradigm shifts to explain its scientific underpinnings.

**Abstract:**

The story of acute promyelocytic leukemia (APL) discovery, physiopathology, and treatment is a unique journey, transforming the most aggressive form of leukemia to the most curable. It followed an empirical route fueled by clinical breakthroughs driving major advances in biochemistry and cell biology, including the discovery of PML nuclear bodies (PML NBs) and their central role in APL physiopathology. Beyond APL, PML NBs have emerged as key players in a wide variety of biological functions, including tumor-suppression and SUMO-initiated protein degradation, underscoring their broad importance. The APL story is an example of how clinical observations led to the incremental development of the first targeted leukemia therapy. The understanding of APL pathogenesis and the basis for cure now opens new insights in the treatment of other diseases, especially other acute myeloid leukemias.

## 1. Introduction

The identification of leukemia (from “leukhemia” meaning “white blood”) is attributed to French Alfred Velpeau and Alfred Donné, as well as British John Hugues Bennett and Prussian Rudolf Virchow, whose early work described symptoms, as well as abnormalities, in the blood composition of several patients [1]. In 1936, Jean Bernard demonstrated that tar coal injection in rat bone marrow caused leukemia development, implying its hematopoietic origin [2]. In the 1950s, leukemia’s medical care was almost nonexistent, despite the early successes of experimentations with irradiation [3] and blood transfusions [4], which induced transient remissions in some cases.

**Figure 1:**
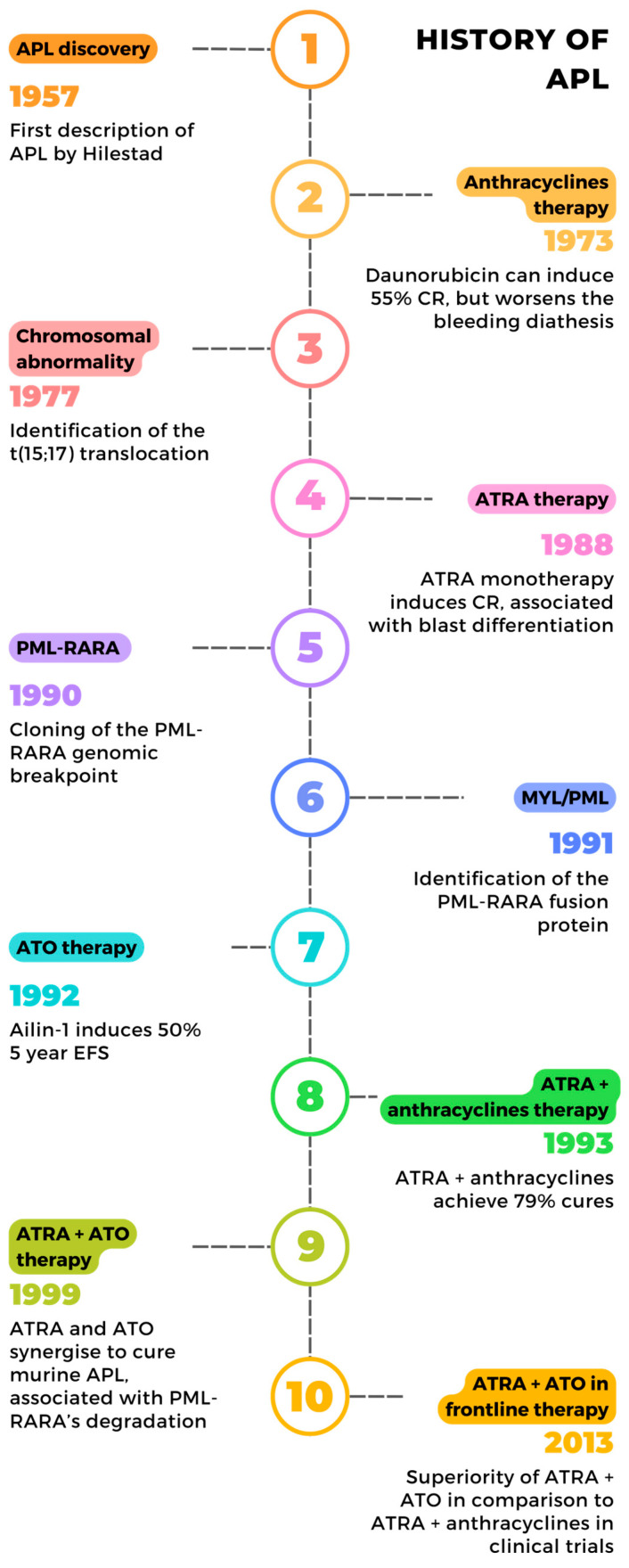
Main milestones in the history of APL cure.

Acute promyelocytic leukemia (APL) was first described by Leif Hillestad in 1957 (Figure 1) in three patients as a subtype of acute myeloid leukemia (AML) with “a very rapid downhill course” requiring a rapid diagnosis and management [5]. Hillestad described blood counts dominated by immature promyelocytes associated with severe bleeding in these patients. These early results were confirmed by the Bernard lab, which described 20 cases of leukemia associated with promyelocytic proliferation and hemorrhagic syndrome [6].

Later studies demonstrated that APL accounts for 10 to 15% of adult AMLs and is nowadays designated under the M3 subtype of the FAB classification, with an incidence of 0.1/100,000 in Western countries [7]. Symptoms include fatigue, fever, weight loss, and infections, as well as bleeding and the formation of blood clots (petechiae, purpura, and ecchymosis). APL patients generally display a low leucocyte count with an invasion of promyelocytic blasts found in the bone marrow and sometimes in the peripheral blood. These blasts display an irregularly shaped nucleus, with large cytoplasmic azurophylic granules and Auer rods, which can aggregate to form “faggots”. Blood diathesis, which most frequently yields hemorrhage in the brain or lung is the main cause of death for APL patients who are not treated on time [8].

## 2. Conventional and Unconventional Therapies

### 2.1. Chemotherapy’s Early Successes

Seminal discoveries were necessary to pave the way for the first APL therapies. Indeed, the development of chemotherapeutic drugs, which would later benefit thousands of patients, ironically originate in the horrors of World War I and World War II and the development of mustard gas. Reports from as early as 1919 described “remarkable changes in the peripheral blood” upon exposure to this poison, with “extreme leukopenia which followed the initial leukocytosis and in severe cases frequently fell below one thousand cells per cubic millimeter” [9]. After WWII, several teams experimented with sulphur mustard for the treatment of Hodgkin’s disease, lymphosarcoma, and leukemia and observed marked regressions in the tumor burden and subsequent brief and incomplete remission in patients [10,11,12]. These encouraging results prompted the development of other systemic anti-cancer drugs like anthracyclines [13]. Among these, daunorubicin monotherapy induced transient remissions in children suffering from leukemia [14].

A major breakthrough in APL therapy came from the Bernard lab, which described the exquisite sensitivity of APL for daunorubicin by inducing complete remissions (CR) in 55% of patients and succeeded in inducing long-term remissions in some of them, surpassing the results obtained with other AMLs [15]. These treatments were further improved by a consolidation with autologous or allogenic bone marrow transplantation [16]. Unfortunately, two-thirds of patients who achieved complete remission (CR) relapsed within the first year, decreasing event-free survival (EFS) to 25–55% [17]. Furthermore, chemotherapy exacerbates bleeding diathesis, thereby increasing the risk of early mortality, underscoring the urgent need to develop safer alternative therapies.

### 2.2. ATRA: Toward a Differentiation Therapy

The 1980s saw the emergence of retinoid-based therapies. The similarities between the histological changes associated with vitamin A deficiency and early cancerous lesions prompted the first assays with retinoids, which demonstrated prophylactic and therapeutic effects in various cancer models in vivo [18]. In the 1960s, capitalizing on all-trans retinoic acid (ATRA) effects on epithelial cells differentiation and proliferation, ATRA therapy was introduced in dermatology for the treatment of psoriasis, ichthyosis, and acne, demonstrating some clinical use [19]. Twenty years later, in the early 1980s, the clinical efficiency of retinoids in leukemia was first demonstrated on a small number of chemotherapy-resistant APL patients, showing some clinical benefits [20,21,22,23,24]. The major breakthrough in APL therapy was published in 1988. The first clinical trial conducted on 20 APL patients demonstrated that ATRA monotherapy can induce long-lasting CR, which is associated with maturation of the leukemic blasts into granulocytes [25], and was later confirmed by other groups [26,27]. Interestingly, ATRA therapy did not prompt severe bleeding, unlike chemotherapy, and rapidly stopped bleeding diathesis.

Despite the dramatic ATRA clinical efficiency, several teams noted that continuous ATRA treatment did not prevent relapses within a median time of 5 months [27,28,29]. This led clinicians to use a combination of ATRA with chemotherapy hoping to combine the long-term remissions induced by chemotherapeutic approaches with the improvement of bleeding diathesis induced by ATRA. A first clinical trial on 26 newly diagnosed APL patients treated with ATRA until CR followed by three courses of anthracyclines evidenced similar CR but a decreased number of relapses in response to the combination therapy when compared with historical data obtained with ATRA alone [30]. Concomitantly, the first European randomized clinical trial was launched, comparing anthracycline therapy with ATRA treatment until CR followed by three courses of anthracyclines. The superiority of ATRA + anthracyclines was such that the trial was prematurely stopped as anthracycline monotherapy was judged unethical, with results as follows: 91% vs. 81% CR and 79% ± 7% vs. 50% ± 9% EFS at 12 months, respectively [17]. Later clinical trials demonstrated that the simultaneous administration of ATRA and anthracyclines followed by a maintenance therapy led to better EFS [31,32,33]. More recent studies of the overall ATRA + anthracyclines therapy successes estimated that this treatment cures up to 80% of APL patients [34]. It should be noted that ATRA treatments led to life-threatening differentiation syndromes in one-third of European APL patients; these were characterized by fever, respiratory distress, unexplained weight gain, peripheral edema, dyspnea with interstitial pulmonary infiltrates, pleuropericardial effusion, hypotension, and acute renal failure [35,36]. Nowadays, differentiation syndromes are managed using corticosteroids, although their mechanisms are not completely understood [37].

### 2.3. Arsenic, a Secular Medicine

These early successes made ATRA-based treatment of APL an example of differentiation therapy as it was thought that terminal granulocytic differentiation elicits cure [38]. The success of APL treatment does not solely rely on ATRA but also involves another unexpected major player that has been utilized in traditional Chinese medicine for a long time, namely, arsenic.

Arsenic (atomic number 33) belongs to the class of transition metals. Arsenic comes from the Greek name “arsenikon”, which means potent. Written testimonies describe its use in a wide variety of conditions since antiquity. The oldest one dates back to Hippocrates, who reports its use to cure ulcers. Colorless, odorless, and tasteless once in solution, arsenic became a very popular poison in medieval Europe and even gained the name “succession powder”. The rehabilitation of arsenic in modern medicine can be attributed to Thomas Fowler, whose eponymous solution was used during the Victorian era to treat various conditions, including syphilis, asthma, chorea, eczema, psoriasis, rheumatism, tuberculosis, and ulcers [39]. The idea that low doses of arsenic could have therapeutic benefits was further supported by the discovery of Austrians “arsenic eaters”, referring to local populations where men, women, and animals consumed arsenic on a daily basis in order to improve physical strength and attractiveness [40]. In 1910, Nobel Prize laureate Paul Ehrlich introduced an organic arsenic-based preparation named salvarsan to treat viral and bacterial infections, such as tuberculosis and syphilis. Derivatives of this preparation were also used to treat infectious diseases, such as African trypanosomiasis, and still is to this day. Interestingly, remissions of what resembles chronic myeloid leukemia in response to Fowler’s solution were reported in several independent studies, first by Prussian physician Berl Klin Wochenschr in 1865 and then by Forkner and Scott from the Boston City Hospital in 1931. An abstract from an 1894 French medical textbook states “the arsenical treatment is to date the one which gave the best clinical results” in the treatment of leukemia [41]. Despite these early hints at arsenic’s therapeutic effects, its high toxicity and the development of new drugs with a higher therapeutic index soon made this secular drug wane.

### 2.4. The Reintroduction of Arsenic in Modern Medicine

Arsenic holds a prominent place in traditional Chinese medicine. In the early 1970s, a Chinese research group from Harbin university tested the clinical response of several cancers to Ailin-1, also called “713”, a mix comprising arsenic trioxide (ATO). They reported that of the 32 treated patients, 21 achieved CR, with a 5-year EFS of 50% and a 10-year EFS of 19% [42]. In 1995, at a meeting of the Chinese Society of Hematology, two independent Chinese groups presented clinical trials with similar results, achieving 73% CR in patients with newly diagnosed APL and 53% CR in relapsed APL patients with 10 mg/day of ATO [41]. One year later, another report described CR in nine out of ten patients with relapsed APL who were treated with ATO [43]. This pioneer study also describes that low concentrations of ATO can induce blast differentiation, while higher doses trigger apoptosis [44]. As Chinese researchers continued to experiment with ATO in the treatment of APL in newly diagnosed and relapsed patients [45], the first clinical trials in western countries were launched in the mid-1990s, reproducing the same results in the induction of CR [46]. Later studies demonstrated that arsenic monotherapy induces not only CR but actual cures in up to 70% of de novo APL patients [47,48].

### 2.5. The ATRA + ATO Synergy

The dramatic success of ATO treatment in APL was immediately of particular interest as patients resistant to ATRA remained sensitive ATO [44,49]. These results begged the question of the efficiency of an ATRA + ATO combination in APL treatment, although ex vivo studies had demonstrated antagonism of the two drugs for differentiation [50]. Yet, pioneer studies demonstrated the overwhelming benefit of the ATRA + ATO combination, which achieved cure in all animals when none of the monotherapies could [51,52]. Several clinical trials confirmed these results and demonstrated the superiority of ATRA + ATO (97% CR) in comparison to other treatments, ATRA + anthracyclines (86% CR) [34,53]. Indeed, the ATRA + ATO combination increases the number of CR, decreases the time necessary to reach remission, and increases EFS [34,54,55,56]. ATRA + ATO therapy is now the gold standard of APL treatment, eliminating the need for additional cytotoxic chemotherapy that can induce bleeding.

Current recommendations for the management of APL patients were reviewed elsewhere [55]. Briefly, diagnosis is based on blood counts, May-Grünwald Giemsa staining indicating an accumulation of promyelocytes, and sometimes, Auer rods in the bone marrow. A rapid confirmation using real-time quantitative polymerase chain reaction (RQ-PCR) should be performed to identify the APL-specific genetic lesion. After ATRA/ATO initiation and consolidation therapies, monitoring of minimal residual disease using RQ-PCR or RT-PCR may be performed on bone marrow samples from high-risk patients.

## 3. Understanding APL

### 3.1. The Discovery of PML-RARA

The first hints at understanding APL’s physiopathology were linked to advances in cytogenetic studies, aiming at identifying chromosomal defects in tissues or cells. In 1976, fluorescence banding performed on bone marrow samples from two APL patients identified the deletion of the long arm of chromosome 17 [57]. The following year, studies confirmed this defect and identified that APL cells harbor a balanced chromosomal translocation between the long arms of chromosomes 15 and 17 [58,59,60].

A few years later, the *RARA* gene was located at the 17q21 position, cytogenetically close to the APL breakpoint location [61]. Exploration of RARA gene structure and expression demonstrated the existence of a specific rearrangement in APL patients [62]. Cloning of the t(15;17) breakpoint in APL, later identified the *RARA* gene fusion to an unknown locus named myeloid leukemia (*MYL*), later renamed promyelocytic leukemia (*PML*) [63,64,65]. The *PML-RARA* fusion gene was shown to encode a fusion protein in all APL patients tested [66,67,68].

Pioneer experiments using gene reporter assays demonstrated that transfected *PML-RARA* in non-hematopoietic cells acts as a dominant negative gene, altering normal *RARA* signaling [66]. These results were later confirmed in myeloid cell lines in which PML-RARA blocked granulocytic differentiation [69]. These experiments raised two questions, one about RARA’s role in myeloid differentiation and the other about the molecular mechanisms underlying PML-RARA’s blockade of granulocytic differentiation.

### 3.2. RARA’s Regulation of Myeloid Differentiation

RARA’s contribution to hematopoiesis was first explored in a *Rara^KO^* mice model. Unexpectedly, granulocytic differentiation was accelerated upon RARA’s excision, but also upon pharmacological doses of ATRA, solely in the presence of RARA [70]. Therefore, RARA slows down granulopoiesis in physiological conditions and is the target of ATRA, mediating its effects on differentiation. In contrast with RARA’s loss, *Rara^403^*, a dominant-negative mutation on the ligand-binding site of the protein that is insensitive to ATRA, triggered profound modifications. This mutant can immortalize hematopoietic progenitors and induces a spontaneous differentiation shift from monocytes to mast cells when expressed in multipotent cell lines [71]. Yet this mutant is very stable, and it was proposed that it may titrate RXR, impeding global signaling of type 2 nuclear receptors. Counterintuitively, the simple overexpression of *Rara^WT^* enables the immortalization of progenitor cells [72] and in the human setting, viral insertions within the RARA gene, inducing its overexpression, impedes differentiation and may induce APL-like symptoms [73,74]. These findings underscore that the ATRA/RARA pair exerts multiple subtle effects on hematopoietic differentiation and self-renewal.

In normal conditions, RARA dimerizes with retinoic X acid receptors (RXRs), binds to retinoic acid response elements, and recruits transcriptional repressors, such as silencing mediator of retinoid and thyroid hormone response (SMRT) or nuclear receptor corepressor (NCOR), maintaining the chromatin in a transcriptional inactive state [75]. The RA binding onto RARA induces a conformational switch of the receptor, whereby co-repressors are replaced by co-activators, thereby inducing the expression of RA-target genes, in particular those implicated in myeloid differentiation [76,77]. In conclusion, RARA acts as a repressor whose activity can be modulated by RA. Increases in RARA’s quantity or impairments in RA-binding result in differentiation blockades and the immortalization of progenitor cells. However, which RARA genes are responsible for APL pathogenesis is still unknown and deserves further investigations.

### 3.3. PML-RARA Impairs the Transcriptional Regulation of Hematopoietic Cells

Since the 1990s, the development of cell lines and several mice models expressing *PML-RARA* cDNA in the myeloid lineage, mimicking APL symptoms has allowed further understanding of APL onset and progression [78,79,80]. Following *PML-RARA*’s initial cloning, first reports described that PML-RARA alters the normal function of RARA in a dominant-negative manner, thus blocking ATRA-induced differentiation at physiological concentrations, like the abovementioned RARA403 mutant [66,69,81].

In comparison to other malignancies, *PML-RARA* is often the sole driver for disease development as demonstrated in several models [78,79,82,83,84]. Moreover, *PML-RARA*’s incidence is almost constant with age, further confirming the idea of a single initiating genetic event [85]. Several cooperating mutations were identified at initial diagnosis or in relapsed APL patients, but their precise role in pathogenesis and resistance is still unclear [84,86]. However several studies have shown that activation of FLT3 signaling blunts RA response in APL or non-APL cells [87,88].

In APL, the PML-RARA oncoprotein binds to DNA through its RARA moiety, acting as a potent transcriptional repressor, insensitive to physiological concentrations of all-trans retinoic acid [89,90], thus blocking myeloid differentiation. Like RARA, PML-RARA dimerizes with RXR [91,92] and efficiently recruits canonical RAR-RXR co-repressors, such as SMRT or NCOR [93], further increasing its transcriptional repressive activity. Moreover, in APL, PML-RARA also transactivates hundreds of non-canonical RARA target genes, in particular, genes coding for chromatin-modifying enzymes or implicated in cell proliferation [92,94,95,96,97]. More importantly, deregulation of retinoic acid signaling is essential for the initiation of APL, as *RARA* fusions are responsible for over 99% of APLs while fusions involving *RARB* or *RARG* are very rare occurrences [98].

Very rare *X-RARA* fusions, not involving *PML*, can also drive APL [98,99]. These APL variants are insensitive to ATO therapy, consistent with PML absence from the fusion proteins. Clinical responses to ATRA therapy were suggested for some patients, although the rareness of these conditions precludes clear-cut answers.

## 4. PML’s Contribution to APL Pathogenesis and Response

### 4.1. PML Nuclear Bodies: Tightly Organized Membrane-Less Compartments

*PML* is the most recurrent partner fused to *RARA* which drives APLs [98]. Immunofluorescence and immunocytochemistry analysis demonstrated that *PML*-transfected cell lines are distributed in a speckled nuclear pattern, called PML nuclear bodies (NBs). In contrast, PML-RARA disorganizes these NBs, which are distributed in a micro-punctuated nuclear pattern, or a cytoplasmic localization in APL blasts expressing both rearranged and non-rearranged PML [100,101]. The disorganization of PML NBs raised inquiries regarding their biological functions and their role in APL development.

PML NBs were first identified through an electron microscopy analysis in the 1960s as electron dense structures in the nucleus of virus-infected rabbit cells [102]. First reports in the 1990s identified the ubiquitous PML expression [66], as well as several splice variants [103]. The following year, immunoelectron and confocal microscopy revealed the core-shell nature of PML NBs, with the PML protein structuring the shell of the body, as well as the presence of several other nuclear proteins in the bodies, including SP-100, but no nucleic acids [104,105]. In APL blasts, PML-RARA disrupts PML NB formation through a direct interaction between the two proteins [100,101].

PML NBs are membrane-less organelles with a diameter of 0.1 to 1 µm that are found in the nucleus of most cells and tissues [106]. Their number varies from 5 to 30 NBs per nucleus, depending on the cell type and the cell cycle [107]. PML belongs to the TRIM (tripartite motif) family of proteins, regrouping proteins harboring a RBCC (RING, B-BOX and coiled-coil), which have the ability to form homo-multimers [108]. Interestingly, many of these proteins display an ubiquitin E3 ligase activity, catalyzing the transfer of the small ubiquitin protein from the E2 ligase to the target substrate [109]. From N- to C-terminus, PML possesses a RING domain, followed by two B-boxes and a coiled-coil domain, which are all essential for PML NB formation [106].

### 4.2. PML Nuclear Bodies: From Structure to Function

After initial PML NB formation by RBCC interactions, PML can recruit UBC9 (the sole SUMO E2 conjugating enzyme) via its RING domain and be SUMOylated [110]. SUMO gets conjugated onto PML on three target lysines in positions K65, K160, and K490 [111,112,113]. PML NBs can then recruit partner proteins through interactions between the SUMO conjugated onto PML’s K160 and the SUMO-interacting motif of client proteins [114,115,116]. PML’s SUMOylation was initially proposed to be mandatory for PML NB formation [117,118]. Later experiments exploring a SUMOylation-deficient mutant (PML-3KR) or a selective inhibitor of the SUMO-activating enzyme proved its non-compulsory nature, as SUMO-deprived PML can still assemble some core-shell NBs [116,119,120]. This reinforces the idea that PML NBs arise from multiple interactions between RBCC motifs [110,121,122,123]. More than 100 client proteins have been identified at PML NBs [115,124]. By concentrating client proteins together with their modification enzymes at their inner core, PML NBs act as hubs for client proteins post-translational modifications, especially SUMOylation and ubiquitination [116,125,126]. Remarkably, a *Pml* knockout in embryonic stem cells phenocopies UBC9 deficiency inducing a transition towards the two-cell-like state, highlighting PML’s central role in the SUMO cascade and downstream biological functions [126,127].

Due to the large number of client proteins modified at PML NBs, these structures have been implicated in the regulation of a wide variety of biological functions, in particular tumor-suppressive functions such as senescence, apoptosis or transcription control [128]. The biological functions linked to PML-NBs were reviewed elsewhere [128]. PML may also assemble in the cytoplasm and a function for cytoplasmic PML bodies was proposed [129].

### 4.3. PML NBs Disruption Block Their Tumor Suppressive Functions

PML tumor suppressive functions have been known since the creation of *Pml^KO^* mice models. These animals are viable and exhibit normal development in the tightly controlled environment of an animal facility but develop more tumors in response to multiple carcinogens [130,131]. Later studies confirmed these results and reported elevated spontaneous tumorigenesis in these animals [132,133,134,135,136]. Nonetheless, some studies on human biopsies from patients further demonstrated that PML NBs are often lost during cancer progression, from various histological origins and that this loss correlates with a poor prognosis [137,138,139].

APL generation in a *Pml^KO^* background only results in an modest increase in leukemia’s incidence and an acceleration of leukemia onset, indicating a tumor-suppressive role of the non-rearranged *Pml* allele [140]. This study was further confirmed with mice models harboring mutations on the RING domain, which block PML NB formation and induces a nucleoplasmic distribution of the PML protein. These mice exhibit a doubling in the rate of leukemia induced by an oncogenic *RARA* fusion [141]. These results demonstrate a role of PML NBs in opposing oncogenic transformation and exclude a role of PML’s diffuse nucleoplasmic fraction in this process.

Early studies focused on the link between PML and senescence control. Precept papers demonstrated that PML opposes oncogene-induced transformation by inducing cell senescence [142,143]. Moreover, mere PML overexpression induces senescence while *Pml^KO^* are resistant to senescence induction [144,145]. Mechanistically, PML regulates senescence both through the Rb/p16 and p53/p21 pathways. On one hand, PML NBs can sequestrate Rb and E2F at nuclear bodies to induce cellular senescence [146,147]. On the other hand, PML NBs recruits p53 regulators, as well as p53 itself, thereby modulating the p53/p21 axis, at least in part through SUMOylation control [148,149,150]. The isoform IV of PML could be the primary effector of this response through a direct interaction with ARF, which in turn, stabilizes UBC9 and SUMOylates TP53 [151]. However, performing a *Pml* knockout in p53-mutated mice elevates the incidence of soft tissue sarcomas and decreased the overall survival in males while increasing the number of osteosarcomas in females, thereby indicating a role of PML in cancer development beyond the context of p53/senescence [136].

### 4.4. PML-Mediated Chromatin Regulation

Another relevant function associated with PML NBs is genome integrity maintenance and transcription control. PML NBs are located at the interchromatin space, but they establish contacts with the chromatin and undergo fission upon nuclear envelope disassembly during cell division [105,152,153]. Moreover, PML plays a role in the transcriptional control both through direct and indirect mechanisms. Indeed, PML associates to chromatin near highly active transcriptional loci, at promotor regions but also in gene-poor regions [153]. These loci do not localize at PML NBs, suggesting a role of the nucleoplasmic fraction of the protein [154,155]. PML NBs also recruit and control the post-translational modifications of numerous proteins at their inner core. This includes chromatin modifiers, such as CBP and HP-1, or chromatin remodelers, such as ATRX [154,156,157,158,159]. This was further confirmed by a recent study, showing that PML NBs can regulate their functions through client protein post-translational modifications, in particular, SUMOylation [126].

### 4.5. PML-Mediated PML-RARA Multimerization

Fusion oncoproteins arise from chromosomal translocations and are observed in ~17% of all human cancers [160]. Many cancers, especially hematological malignancies, are characterized by fusions between genes encoding multimerization domains and chromatin-binding proteins, driving aberrant downstream gene expression by increasing DNA binding affinity, allowing association with new targets [161]. Interestingly, some other members of the TRIM family are involved in cancer development, some of which are due to chromosomal translocations like TRIM24/TIF1a, TRIM27/RFP, and TRIM33/TIF1g, whose RBCC motifs play a critical role in cell transformation [162,163].

The fusion of PML’s coiled-coil domain to RARA is sufficient to transform hematopoietic progenitors in vivo, while only the overexpression of *RARA* induces an equivalent phenotype ex vivo [72,164]. Conversely, mutations on PML RING or B1 domains abrogating multimerization impede the immortalization of hematopoietic progenitors, while less stringent mutations that only partially block PML self-assembly do not [91,110,122,149,165]. Moreover, PML fusion to RARA drastically decreases its mobility at microspeckles [123], and all RARA oncogenic fusions display multimerization domains [98]. Yet, the fusion of synthetic dimerization domains to RARA only allows incomplete transformation in vivo, suggesting that some features of PML itself may be necessary [166,167]. Altogether, these elements point to a crucial role of PML-driven multimerization of PML-RARA in APL onset and development.

### 4.6. Recruitment of Partner Proteins on PML-RARA

PML’s SUMOylated lysine K160, which is responsible for partner protein recruitment, is conserved in PML-RARA [112,116]. The SUMOylation of PML-RARA through K160 is mandatory for optimal leukemic transformation [168]. The fusion of the DAXX transcriptional repressor to the *PML-RARA^K160R^* mutant oncoprotein restores the oncogenic transformation, pointing to a role of PML-partner proteins-mediated transcriptional regulation in APL [168]. Thus, PML-dependent recruitment of partner proteins may contribute to transformation. The RXR protein is essential for leukemic transformation through the formation of a RARA-RXR dimer, and its PML-RARA-dependent SUMOylation may favor this process [91,169]. The N-COR transcriptional co-repressor is also associated to PML-RARA, and its SUMOylation, which might be promoted by PML-RARA, favors its transcriptional repressive activities [170,171]. Altogether, the recruitment/modification of PML partner proteins could further control the repression of target genes implicated in hematopoietic differentiation and self-renewal [92,96,97,170,172,173].

### 4.7. PML and Leukemia Initiating Cells

PML is never lost at the genetic level and only rarely mutated in APL [84,149,174], despite a mild selective advantage of *Pml^KO^* blasts over their *Pml^WT^* counterpart, pointing to a context-dependent, pro-tumoral role of PML [140]. Studies focused on the control of embryonic stem cells (ESCs) self-renewal and differentiation identified a central role of PML in this process. PML promotes ESC cellular cycle progression, as well as lineage, and its down-regulation alleviates the reprograming of mouse embryonic fibroblasts into IPSCs [175]. PML does so by controlling SUMOylation of numerous partner proteins, especially members of the KAP1 complex, as well as that of the transcription factor DPPA2, modulating the transcriptional activity and 3D genome organization [126,176]. In AML patients samples, only a rare subset of immature cells, leukemia initiating cells (LICs), are able to reestablish the disease in vivo [177,178]. These LICs display many features of hematopoietic stem cells and are enriched in minimal residual disease after treatment, contributing to relapses [179,180]. In chronic myeloid leukemia, PML is highly expressed in LICs and plays a central role in their maintenance [181]. Mechanistically, PML decreases the activity mTOR pathway by inducing the expression of the PPARg master regulator and by increasing fatty acid metabolism, thereby allowing stem cell asymmetric division [182]. Similar mechanisms have since been described in breast and ovarian cancers and could play a role in APL [183,184,185,186].

## 5. Understanding APL Cure

### 5.1. ATO Drives PML-RARA Degradation

The first studies focused on PML subcellular localization both in APL and non-APL context reported that ATO induces aggregation of both PML and PML-RARA at short time points [101,187]. Subsequent research demonstrated that over time, ATO induces their degradation, even at minimal doses of 10^−8^ M, thereby prompting APL blast’s clearance [49,187]. This degradation-based therapy of APL contrasted with the classical view of ATRA-induced differentiation therapy.

Mechanistically, ATO has a dual effect. It can induce oxidative stress through mitochondria toxicity by poisoning the mitochondrial respiration complexes or by scavenging antioxidant proteins [188]. It can also directly bind to free cysteine residues due to its high affinity for sulfhydryl groups. PML is a cysteine-rich, oxidation-prone protein, and oxidative stress induced by multiple drugs, like paracetamol, induces NB assembly in vivo, similar to ATO [113,189,190].

**Figure 2:**
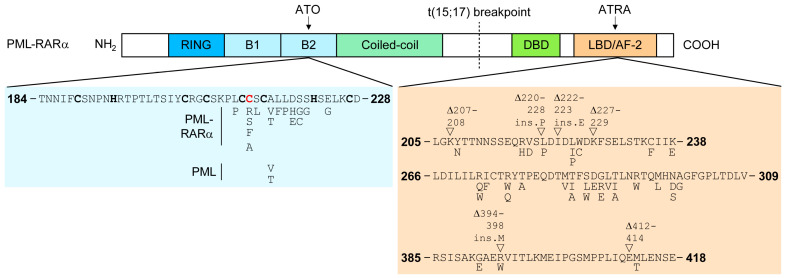
Genetic mutations of PML-RARA identified in therapy-resistant patients. Amino acids coordinating the zinc in PML’s B2 box are indicated in bold font. The critical C213 cysteine responsible for arsenic-binding onto PML is indicated in bold red font.

ATO-induced PML NB formation results from its direct binding onto the PML protein. Indeed, point mutations around a di-cysteine motif in PML’s B2 box were identified in ATO-resistant APL patients (Figure 2) [84,149]. Moreover, experiments performed with fluorescent arsenic, which only fluoresces when bound to proteins, demonstrated direct arsenic binding onto the shell of PML NBs [189]. A recent study from our lab demonstrated that the homo-trimeric assembly of PML B2 regroups three free cysteines (C213) in the center of the structure in what resembles an arsenic-binding site [123,189]. Arsenic binding onto this cysteine rheostat induces a “gelling” of PML at nuclear bodies, likely favoring the recruitment and enzymatic efficiency of the SUMO machinery. Subsequently, ATO induces PML-RARA hyper-SUMOylation on PML’s lysine K160, subsequent RNF4 ubiquitin ligase, and ultimately, proteasomal degradation [112,114,191]. Recent studies proposed that other ubiquitin ligases, such as RNF111/Arkadia, may be involved in ubiquitin-dependent PML degradation [192,193]. The p90 segregase also removes the ubiquitinated PML protein from the NB prior to its proteasomal degradation [194].

Therapy response is compromised in APL blasts harboring a PML mutation on the non-rearranged allele, which hinders NB formation, thereby stressing PML’s role in this context [84,141]. PML-RARA’s degradation restores normal PML–PML interaction and arsenic-binding onto normal PML also enforces NB formation [187]. This leads to a massive SUMOylation of client proteins, including many p53 regulators and p53 itself [116,126], thereby restoring a p53 checkpoint and inducing a TP53 response exhibiting features of senescence responsible for the clearance of APL blasts [148].

### 5.2. Revisiting ATRA Treatment of APL: Is It a Differentiation Therapy?

The clinical response of APL patients to ATRA monotherapy is short despite reaching terminal granulocytic differentiation and the restoration of normal bone marrow. The requirement to combine ATRA with chemotherapy to maintain remission contradicts the classical view of differentiation-driven therapy [195,196]. In most cases, patients relapsed quickly, sometimes due to acquired mutations in on the ligand-binding domain of RARA [86,197].

The demonstration of ATO-mediated PML-RARA degradation gave a new basis of reasoning to explain ATRA’s therapeutic effects. Indeed, high doses of ATRA trigger a negative feedback loop, inducing PML-RARA’s proteasomal degradation through its RARA moiety [198,199], suggesting that ATRA therapy could also be a degradation-based therapy. Indeed, ATO treatment also prompts the differentiation of APL blasts in vitro and in vivo [44]. This suggests that alleviating PML-RARA’s transcriptional blockade through its degradation suffices to trigger this process [169].

The respective roles of transcriptional activation and degradation were debated until the analysis of synthetic retinoids, which can potently activate RARA- or PML-RARA-dependent transcription but failed to induce RARA degradation. APL cells treated with these drugs reached terminal differentiation, but some blasts retained PML-RARA expression and reinitiated APL. Thus, complete PML-RARA degradation is necessary for APL [200]. Similarly, only high doses of ATRA and active proteolysis allows for the complete clearance of APL [201]. In contrast, low ATRA doses only induce transient remissions despite triggering terminal differentiation. Recently, in vivo studies have demonstrated that the differentiation of *p53^KO^* AML cells can be reversible, thereby highlighting the necessity to eliminate the leukemic cells [202]. Actually, in APL patients, liposomal ATRA, which allows prolonged high plasma concentrations, drives some long-term remissions [203,204]. In summary, ATRA-induced PML-RARA degradation, rather than its sole impact on transcription, is required for its efficacy in curing APL.

Interestingly, the targeting of PML by ATO drives LIC eradication by inducing full PML-RARA degradation and p53-mediated senescence, resulting in low relapses rates [148,201]. This phenomenon is absent in non-PML driven APLs, confirming the pivotal role of PML [55].

## 6. Conclusions and Future Perspectives

ATO targeting of PML revolutionized our view of APL cure from a differentiation-based therapy to a degradation-based therapy. The ATRA + ATO combination is the frontline therapy, which nowadays, cures 97% of patients. The comprehension of the molecular mechanisms involved in APL pathogenesis and cure unraveled some features that could be targeted in other conditions. Indeed, PML protein is lost in many cancers from various histological origins, while its mRNA is still detected, suggesting a post-translational control-inducing PML degradation [132,138,205]. Several therapeutic strategies already gave promising results. In adult T-cell leukemia, PML NB formation triggered by interferon and ATO induces oncogenic TAX SUMO-dependent degradation and improves patients’ prognosis when used as a consolidation therapy with chemotherapy [206,207,208]. In AML-harboring NPM1c mutations, retinoic acid stabilizes PML through Pin1 inhibition, inducing TP53 activities and chemotherapy response [209,210]. In the same model, actinomycin D induces ROS production from the mitochondria, inducing PML NB formation and TP53 activation in vivo [211]. In a murine model of glioblastoma, the induction of PML expression by interferon improves the therapeutic effects of temozolomide by activating p73-mediated apoptosis [212]. In myeloproliferative neoplasia, interferon and ATO induce PML-dependent LIC’s senescence and eradicates the disease in a mouse model [213].

A converse strategy would consist in abolishing PML NBs through the induction of PML degradation. In a glioma xenograft mouse model, ATO-induced PML degradation triggers a decrease in c-Myc and inhibits tumor growth [214]. The induction of PML degradation triggers a similar response in a mouse model of triple-negative breast cancer by inducing a p27-mediated senescence [186]. Moreover, recent results on patients-derived cells demonstrated a potential clinical application of ATO-induced PML degradation in pediatric glioma [215].

Future studies will determine if these results can translate in the clinic. Finally, the recent understanding of the molecular mechanisms of ATO binding onto PML B2 could lead to the development of new therapeutics inducing PML NB formation, or disorganization, with a lesser toxicity than the original drug.

The story of APL cure also highlighted the high clinical potential of ATRA, which could be of significant use in other pathologies. Indeed, deregulated retinoic acid signaling was reported in other cancers (human breast and murine liver) and some non-APL AMLs [216,217,218]. Although these deregulations are diverse, AMLs expressing high levels of RARA are predisposed to an exquisite sensitivity to ATRA which induces differentiation and loss of proliferation in patients-derived xenografts models [218]. Clinical trials led in AML patients unfit for chemotherapy demonstrated that ATRA addition to DNA-hypomethylating agents resulted in higher remission rates and meaningful survival extensions [219,220]. Moreover, in AMLs without RARA rearrangements, which were treated with pharmacological doses of ATRA following an initial suspicion of APL, the exhibit acquired RARA mutations in its LBD [221]. This implies a selective advantage associated with the inhibition of ATRA-induced RARA activation. ATRA-treatment of APL, therefore, may open new opportunities for managing otherwise incurable AMLs. Future studies, using the lessons from the APL saga, will hopefully lead to new cures in other pathologies.

## Data Availability

Data will be made available by the corresponding author upon request.

## References

[1] Kampen K.R. (2012). The Discovery and Early Understanding of Leukemia. Leuk. Res..

[2] Bernard J. (1936). Polyglobulies et Leucémies Provoquées Par Des Injections Intramédullaires de Goudron. Ph.D. Thesis.

[3] Lederman M. (1981). The Early History of Radiotherapy: 1895–1939. Int. J. Radiat. Oncol. Biol. Phys..

[4] Bessis M., Bernard J. (1947). Remarkable results of the exsanguino-transfusion treatment of a case of acute leukemia. Bull. Mem. Soc. Med. Hop. Paris.

[5] Hillestad L.K. (1957). Acute Promyelocytic Leukemia. Acta Med. Scand..

[6] Bernard J., Mathe G., Boulay J., Ceoard B., Chome J. (1959). Acute promyelocytic leukemia: A study made on 20 cases. Schweiz. Med. Wochenschr..

[7] Sant M., Allemani C., Tereanu C., De Angelis R., Capocaccia R., Visser O., Marcos-Gragera R., Maynadié M., Simonetti A., Lutz J.-M. (2010). Incidence of Hematologic Malignancies in Europe by Morphologic Subtype: Results of the HAEMACARE Project. Blood.

[8] Rickles F.R., Falanga A., Montesinos P., Sanz M.A., Brenner B., Barbui T. (2007). Bleeding and Thrombosis in Acute Leukemia: What Does the Future of Therapy Look Like?. Thromb. Res..

[9] Krumbhaar E.B., Krumbhaar H.D. (1919). The Blood and Bone Marrow in Yelloe Cross Gas (Mustard Gas) Poisoning: Changes Produced in the Bone Marrow of Fatal Cases. J. Med. Res..

[10] Goodman L.S. (1946). Nitrogen Mustard Therapy: Use of Methyl-Bis(Beta-Chloroethyl)Amine Hydrochloride and Tris(Beta-Chloroethyl)Amine Hydrochloride for Hodgkin’s Disease, Lymphosarcoma, Leukemia and Certain Allied and Miscellaneous Disorders. JAMA.

[11] Gilman A. (1946). Therapeutic Applications of Chemical Warfare Agents. Fed. Proc..

[12] Gilman A., Philips F.S. (1946). The Biological Actions and Therapeutic Applications of the B-Chloroethyl Amines and Sulfides. Science.

[13] Grein A., Spalla C., di Marco A., Canevazzi G. (1963). Descrizione e Classificazione Di Un Attinomicete (Streptomyces Peucetius Sp. Nova). Produttore Di Una Sostanza Ad Attivit Antitumorale: La Daunomicina. Giorn. Microbiol..

[14] Tan C., Tasaka H., Yu K.-P., Murphy M.L., Karnofsky D.A. (1967). Daunomycin, an Antitumor Antibiotic, in the Treatment of Neoplastic Disease.Clinical Evaluation with Special Reference to Childhood Leukemia. Cancer.

[15] Bernard J., Weil M., Boiron M., Jacquillat C., Flandrin G., Gemon M.F. (1973). Acute Promyelocytic Leukemia: Results of Treatment by Daunorubicin. Blood.

[16] Mandelli F., Labopin M., Granena A., Iriondo A., Prentice G., Bacigalupo A., Sierra J., Meloni G., Frassoni F., Goldman J. (1994). European Survey of Bone Marrow Transplantation in Acute Promyelocytic Leukemia (M3). Working Party on Acute Leukemia of the European Cooperative Group for Bone Marrow Transplantation (EMBT). Bone Marrow Transpl..

[17] Fenaux P., Le Deley M.C., Castaigne S., Archimbaud E., Chomienne C., Link H., Guerci A., Duarte M., Daniel M.T., Bowen D. (1993). Effect of All Transretinoic Acid in Newly Diagnosed Acute Promyelocytic Leukemia. Results of a Multicenter Randomized Trial. European APL 91 Group. Blood.

[18] Bollag W. (1979). Retinoids and Cancer. Cancer Chemother. Pharmacol..

[19] Liang C., Qiao G., Liu Y., Tian L., Hui N., Li J., Ma Y., Li H., Zhao Q., Cao W. (2021). Overview of All-Trans-Retinoic Acid (ATRA) and Its Analogues: Structures, Activities, and Mechanisms in Acute Promyelocytic Leukaemia. Eur. J. Med. Chem..

[20] Daenen S., Vellenga E., van Dobbenburgh O.A., Halie M.R. (1986). Retinoic Acid as Antileukemic Therapy in a Patient with Acute Promyelocytic Leukemia and Aspergillus Pneumonia. Blood.

[21] Flynn P.J., Miller W.J., Weisdorf D.J., Arthur D.C., Brunning R., Branda R.F. (1983). Retinoic Acid Treatment of Acute Promyelocytic Leukemia: In Vitro and in Vivo Observations. Blood.

[22] Fontana J.A., Rogers J.S., Durham J.P. (1986). The Role of 13 Cis-Retinoic Acid in the Remission Induction of a Patient with Acute Promyelocytic Leukemia. Cancer.

[23] Nilsson B. (1984). Probable in Vivo Induction of Differentiation by Retinoic Acid of Promyelocytes in Acute Promyelocytic Leukaemia. Br. J. Haematol..

[24] Sampi K., Honma Y., Hozumi M., Sakurai M. (1985). Discrepancy between In-Vitro and in-Vivo Inductions of Differentiation by Retinoids of Human Acute Promyelocytic Leukemia Cells in Relapse. Leuk. Res..

[25] Huang M., Ye Y., Chen S., Chai J., Lu J., Zhoa L., Gu L., Wang Z. (1988). Use of All-Trans Retinoic Acid in the Treatment of Acute Promyelocytic Leukemia. Blood.

[26] Degos L., Chomienne C., Daniel M.T., Berger R., Dombret H., Fenaux P., Castaigne S. (1990). Treatment of First Relapse in Acute Promyelocytic Leukaemia with All-Trans Retinoic Acid. Lancet.

[27] Warrell R.P., Frankel S.R., Miller W.H., Scheinberg D.A., Itri L.M., Hittelman W.N., Vyas R., Andreeff M., Tafuri A., Jakubowski A. (1991). Differentiation Therapy of Acute Promyelocytic Leukemia with Tretinoin (All-Trans-Retinoic Acid). N. Engl. J. Med..

[28] Castaigne S., Chomienne C., Daniel M.T., Ballerini P., Berger R., Fenaux P., Degos L. (1990). All-Trans Retinoic Acid as a Differentiation Therapy for Acute Promyelocytic Leukemia. I. Clinical Results. Blood.

[29] Chen Z.X., Xue Y.Q., Zhang R., Tao R.F., Xia X.M., Li C., Wang W., Zu W.Y., Yao X.Z., Ling B.J. (1991). A Clinical and Experimental Study on All-Trans Retinoic Acid-Treated Acute Promyelocytic Leukemia Patients. Blood.

[30] Fenaux P., Castaigne S., Dombret H., Archimbaud E., Duarte M., Morel P., Lamy T., Tilly H., Guerci A., Maloisel F. (1992). All-Transretinoic Acid Followed by Intensive Chemotherapy Gives a High Complete Remission Rate and May Prolong Remissions in Newly Diagnosed Acute Promyelocytic Leukemia: A Pilot Study on 26 Cases. Blood.

[31] Tallman M.S., Andersen J.W., Schiffer C.A., Appelbaum F.R., Feusner J.H., Ogden A., Shepherd L., Willman C., Bloomfield C.D., Rowe J.M. (1997). All- *Trans* -Retinoic Acid in Acute Promyelocytic Leukemia. N. Engl. J. Med..

[32] Fenaux P., Chastang C., Chevret S., Sanz M., Dombret H., Archimbaud E., Fey M., Rayon C., Huguet F., Sotto J.J. (1999). A Randomized Comparison of All Transretinoic Acid (ATRA) Followed by Chemotherapy and ATRA plus Chemotherapy and the Role of Maintenance Therapy in Newly Diagnosed Acute Promyelocytic Leukemia. The European APL Group. Blood.

[33] Degos L., Dombret H., Chomienne C., Daniel M., Miclea J., Chastang C., Castaigne S., Fenaux P. (1995). All-Trans-Retinoic Acid as a Differentiating Agent in the Treatment of Acute Promyelocytic Leukemia. Blood.

[34] Lo-Coco F., Avvisati G., Vignetti M., Thiede C., Orlando S.M., Iacobelli S., Ferrara F., Fazi P., Cicconi L., Di Bona E. (2013). Retinoic Acid and Arsenic Trioxide for Acute Promyelocytic Leukemia. N. Engl. J. Med..

[35] Frankel S.R., Eardley A., Lauwers G., Weiss M., Warrell R.P. (1992). The “Retinoic Acid Syndrome” in Acute Promyelocytic Leukemia. Ann. Intern. Med..

[36] Sanz M.A., Montesinos P. (2014). How We Prevent and Treat Differentiation Syndrome in Patients with Acute Promyelocytic Leukemia. Blood.

[37] Reyhanoglu G., Hughes B., King K.E., Cambridge R. (2020). Differentiation Syndrome, a Side Effect from the Therapy of Acute Promyelocytic Leukemia. Cureus.

[38] Warrell R.P., de The H., Wang Z.-Y., Degos L. (1993). Acute Promyelocytic Leukemia. N. Engl. J. Med..

[39] Haller J.S. (1975). Therapeutic Mule: The Use of Arsenic in the Nineteenth Century Materia Medica. Pharm. Hist..

[40] Zhu J., Chen Z., Lallemand-Breitenbach V., de Thé H. (2002). How Acute Promyelocytic Leukaemia Revived Arsenic. Nat. Rev. Cancer.

[41] Degos L. (2003). The History of Acute Promyelocytic Leukaemia. Br. J. Haematol..

[42] Sun H.D., Ma L., Hu X.C., Zhang T.D. (1992). Ai-Lin I Treated 32 Cases of Acute Promyelocytic Leukemia. Chin. J Integrat. Chin. West Med..

[43] Shen Z.-X., Chen G.-Q., Ni J.-H., Li X.-S., Xiong S.-M., Qiu Q.-Y., Zhu J., Tang W., Sun G.-L., Yang K.-Q. (1997). Use of Arsenic Trioxide (As_2_O_3_) in the Treatment of Acute Promyelocytic Leukemia (APL): II. Clinical Efficacy and Pharmacokinetics in Relapsed Patients. Blood.

[44] Chen G.Q., Shi X.G., Tang W., Xiong S.M., Zhu J., Cai X., Han Z.G., Ni J.H., Shi G.Y., Jia P.M. (1997). Use of Arsenic Trioxide (As_2_O_3_) in the Treatment of Acute Promyelocytic Leukemia (APL): I. As_2_O_3_ Exerts Dose-Dependent Dual Effects on APL Cells. Blood.

[45] Niu C., Yan H., Yu T., Sun H.P., Liu J.X., Li X.S., Wu W., Zhang F.Q., Chen Y., Zhou L. (1999). Studies on Treatment of Acute Promyelocytic Leukemia with Arsenic Trioxide: Remission Induction, Follow-up, and Molecular Monitoring in 11 Newly Diagnosed and 47 Relapsed Acute Promyelocytic Leukemia Patients. Blood.

[46] Soignet S.L., Maslak P., Wang Z.-G., Jhanwar S., Calleja E., Dardashti L.J., Corso D., DeBlasio A., Gabrilove J., Scheinberg D.A. (1998). Complete Remission after Treatment of Acute Promyelocytic Leukemia with Arsenic Trioxide. N. Engl. J. Med..

[47] Mathews V., George B., Chendamarai E., Lakshmi K.M., Desire S., Balasubramanian P., Viswabandya A., Thirugnanam R., Abraham A., Shaji R.V. (2010). Single-Agent Arsenic Trioxide in the Treatment of Newly Diagnosed Acute Promyelocytic Leukemia: Long-Term Follow-Up Data. J. Clin. Oncol..

[48] Ghavamzadeh A., Alimoghaddam K., Rostami S., Ghaffari S.H., Jahani M., Iravani M., Mousavi S.A., Bahar B., Jalili M. (2011). Phase II Study of Single-Agent Arsenic Trioxide for the Front-Line Therapy of Acute Promyelocytic Leukemia. J. Clin. Oncol..

[49] Chen G., Zhu J., Shi X., Ni J., Zhong H., Si G., Jin X., Tang W., Li X., Xong S. (1996). In Vitro Studies on Cellular and Molecular Mechanisms of Arsenic Trioxide (As_2_O_3_) in the Treatment of Acute Promyelocytic Leukemia: As_2_O_3_ Induces NB4 Cell Apoptosis with Downregulation of Bcl-2 Expression and Modulation of PML-RAR Alpha/PML Proteins. Blood.

[50] Shao W., Fanelli M., Ferrara F.F., Riccioni R., Rosenauer A., Davison K., Lamph W.W., Waxman S., Pelicci P.G., Lo Coco F. (1998). Arsenic Trioxide as an Inducer of Apoptosis and Loss of PML/RARα Protein in Acute Promyelocytic Leukemia Cells. JNCI J. Natl. Cancer Inst..

[51] Lallemand-Breitenbach V., Guillemin M.-C., Janin A., Daniel M.-T., Degos L., Kogan S.C., Michael Bishop J., de Thé H. (1999). Retinoic Acid and Arsenic Synergize to Eradicate Leukemic Cells in a Mouse Model of Acute Promyelocytic Leukemia. J. Exp. Med..

[52] Rego E.M., He L.-Z., Warrell R.P., Wang Z.-G., Pandolfi P.P. (2000). Retinoic Acid (RA) and As_2_O_3_ Treatment in Transgenic Models of Acute Promyelocytic Leukemia (APL) Unravel the Distinct Nature of the Leukemogenic Process Induced by the PML-RARα and PLZF-RARα Oncoproteins. Proc. Natl. Acad. Sci. USA.

[53] Estey E., Garcia-Manero G., Ferrajoli A., Faderl S., Verstovsek S., Jones D., Kantarjian H. (2006). Use of All-Trans Retinoic Acid plus Arsenic Trioxide as an Alternative to Chemotherapy in Untreated Acute Promyelocytic Leukemia. Blood.

[54] Wang H., Chen X., Wang B., Rong Z., Qi H., Chen H. (2011). The Efficacy and Safety of Arsenic Trioxide with or without All-Trans Retinoic Acid for the Treatment of Acute Promyelocytic Leukemia: A Meta-Analysis. Leuk. Res..

[55] Sanz M.A., Fenaux P., Tallman M.S., Estey E.H., Löwenberg B., Naoe T., Lengfelder E., Döhner H., Burnett A.K., Chen S.-J. (2019). Management of Acute Promyelocytic Leukemia: Updated Recommendations from an Expert Panel of the European LeukemiaNet. Blood.

[56] Long Z.-J., Hu Y., Li X.-D., He Y., Xiao R.-Z., Fang Z.-G., Wang D.-N., Liu J.-J., Yan J.-S., Huang R.-W. (2014). ATO/ATRA/Anthracycline-Chemotherapy Sequential Consolidation Achieves Long-Term Efficacy in Primary Acute Promyelocytic Leukemia. PLoS ONE.

[57] Golomb H.M., Rowley J., Vardiman J., Baron J., Locker G., Krasnow S. (1976). Partial Deletion of Long Arm of Chromosome 17: A Specific Abnormality in Acute Promyelocytic Leukemia?. Arch. Intern. Med..

[58] Rowley J.D., Golomb H.M., Vardiman J., Fukuhara S., Dougherty C., Potter D. (1977). Further Evidence for a Non-Random Chromosomal Abnormality in Acute Promyelocytic Leukemia. Int. J. Cancer.

[59] Rowley J.D., Golomb H.M., Dougherty C. (1977). 15/17 Translocation, a Consistent Chromosomal Change in Acute Promyelocytic Leukaemia. Lancet.

[60] Larson R.A., Kondo K., Vardiman J.W., Butler A.E., Golomb H.M., Rowley J.D. (1984). Evidence for a 15; 17 Translocation in Every Patient with Acute Promyelocytic Leukemia. Am. J. Med..

[61] Mattei M.-G., Petkovich M., Mattei J.-F., Brand N., Chambon P. (1988). Mapping of the Human Retinoic Acid Receptor to the Q21 Band of Chromosome 17. Hum. Genet..

[62] Chomienne C., Ballerini P., Balitrand N., Huang M.E., Krawice I., Castaigne S., Fenaux P., Tiollais P., Dejean A., Degos L. (1990). The Retinoic Acid Receptor Alpha Gene Is Rearranged in Retinoic Acid-Sensitive Promyelocytic Leukemias. Leukemia.

[63] de Thé H., Chomienne C., Lanotte M., Degos L., Dejean A. (1990). The t(15;17) Translocation of Acute Promyelocytic Leukaemia Fuses the Retinoic Acid Receptor α Gene to a Novel Transcribed Locus. Nature.

[64] Borrow J., Goddard A., Sheer D., Solomon E. (1990). Molecular Analysis of Acute Promyelocytic Leukemia Breakpoint Cluster Region on Chromosome 17. Science.

[65] Longo L., Pandolfi P.P., Biondi A., Rambaldi A., Mencarelli A., Lo Coco F., Diverio D., Pegoraro L., Avanzi G., Tabilio A. (1990). Rearrangements and Aberrant Expression of the Retinoic Acid Receptor Alpha Gene in Acute Promyelocytic Leukemias. J. Exp. Med..

[66] de Thé H., Lavau C., Marchio A., Chomienne C., Degos L., Dejean A. (1991). The PML-RARα Fusion mRNA Generated by the t(15;17) Translocation in Acute Promyelocytic Leukemia Encodes a Functionally Altered RAR. Cell.

[67] Goddard A.D., Borrow J., Freemont P.S., Solomon E. (1991). Characterization of a Zinc Finger Gene Disrupted by the t(15;17) in Acute Promyelocytic Leukemia. Science.

[68] Kakizuka A., Miller W.H., Umesono K., Warrell R.P., Frankel S.R., Murty V.V.V.S., Dmitrovsky E., Evans R.M. (1991). Chromosomal Translocation t(15;17) in Human Acute Promyelocytic Leukemia Fuses RARα with a Novel Putative Transcription Factor, PML. Cell.

[69] Rousselot P., Hardas B., Patel A., Guidez F., Gäken J., Castaigne S., Dejean A., de Thé H., Degos L., Farzaneh F. (1994). The PML-RAR Alpha Gene Product of the t(15;17) Translocation Inhibits Retinoic Acid-Induced Granulocytic Differentiation and Mediated Transactivation in Human Myeloid Cells. Oncogene.

[70] Kastner P., Lawrence H.J., Waltzinger C., Ghyselinck N.B., Chambon P., Chan S. (2001). Positive and Negative Regulation of Granulopoiesis by Endogenous RARα. Blood.

[71] Collins S. (2002). The Role of Retinoids and Retinoic Acid Receptors in Normal Hematopoiesis. Leukemia.

[72] Du C., Redner R.L., Cooke M.P., Lavau C. (1999). Overexpression of Wild-Type Retinoic Acid Receptor Alpha (RARalpha) Recapitulates Retinoic Acid-Sensitive Transformation of Primary Myeloid Progenitors by Acute Promyelocytic Leukemia RARalpha-Fusion Genes. Blood.

[73] Astolfi A., Masetti R., Indio V., Bertuccio S.N., Messelodi D., Rampelli S., Leardini D., Carella M., Serravalle S., Libri V. (2021). Torque Teno Mini Virus as a Cause of Childhood Acute Promyelocytic Leukemia Lacking PML/RARA Fusion. Blood.

[74] Chen X., Wang F., Zhou X., Zhang Y., Cao P., Ma X., Yuan L., Fang J., Liu M., Liu M. (2022). Torque Teno Mini Virus Driven Childhood Acute Promyelocytic Leukemia: The Third Case Report and Sequence Analysis. Front. Oncol..

[75] Privalsky M.L. (2004). The Role of Corepressors in Transcriptional Regulation by Nuclear Hormone Receptors. Annu. Rev. Physiol..

[76] Huang P., Chandra V., Rastinejad F. (2014). Retinoic Acid Actions through Mammalian Nuclear Receptors. Chem. Rev..

[77] le Maire A., Teyssier C., Balaguer P., Bourguet W., Germain P. (2019). Regulation of RXR-RAR Heterodimers by RXR- and RAR-Specific Ligands and Their Combinations. Cells.

[78] Brown D., Kogan S., Lagasse E., Weissman I., Alcalay M., Pelicci P.G., Atwater S., Bishop J.M. (1997). A PMLRARα Transgene Initiates Murine Acute Promyelocytic Leukemia. Proc. Natl. Acad. Sci. USA.

[79] Grisolano J.L., Wesselschmidt R.L., Pelicci P.G., Ley T.J. (1997). Altered Myeloid Development and Acute Leukemia in Transgenic Mice Expressing PML-RAR Alpha under Control of Cathepsin G Regulatory Sequences. Blood.

[80] Lanotte M., Martin-Thouvenin V., Najman S., Balerini P., Valensi F., Berger R. (1991). NB4, a Maturation Inducible Cell Line with t(15;17) Marker Isolated from a Human Acute Promyelocytic Leukemia (M3). Blood.

[81] Collins S.J., Robertson K.A., Mueller L. (1990). Retinoic Acid-Induced Granulocytic Differentiation of HL-60 Myeloid Leukemia Cells Is Mediated Directly through the Retinoic Acid Receptor (RAR-α). Mol. Cell. Biol..

[82] Kogan S.C., Pandolfi P.P., Vogt P.K. (2007). Mouse Models of Acute Promyelocytic Leukemia. Acute Promyelocytic Leukemia.

[83] Welch J.S., Yuan W., Ley T.J. (2008). Expression of PML-RARα by the Murine PML Locus Leads to Myeloid Self-Renewal, Clonal Expansion and Morphologic Promyelocytic Leukemia. Blood.

[84] Lehmann-Che J., Bally C., Letouzé E., Berthier C., Yuan H., Jollivet F., Ades L., Cassinat B., Hirsch P., Pigneux A. (2018). Dual Origin of Relapses in Retinoic-Acid Resistant Acute Promyelocytic Leukemia. Nat. Commun..

[85] Vickers M., Jackson G., Taylor P. (2000). The Incidence of Acute Promyelocytic Leukemia Appears Constant over Most of a Human Lifespan, Implying Only One Rate Limiting Mutation. Leukemia.

[86] Madan V., Shyamsunder P., Han L., Mayakonda A., Nagata Y., Sundaresan J., Kanojia D., Yoshida K., Ganesan S., Hattori N. (2016). Comprehensive Mutational Analysis of Primary and Relapse Acute Promyelocytic Leukemia. Leukemia.

[87] Ma H.S., Greenblatt S.M., Shirley C.M., Duffield A.S., Bruner J.K., Li L., Nguyen B., Jung E., Aplan P.D., Ghiaur G. (2016). All-Trans Retinoic Acid Synergizes with FLT3 Inhibition to Eliminate FLT3/ITD+ Leukemia Stem Cells in Vitro and in Vivo. Blood.

[88] Esnault C., Rahmé R., Rice K.L., Berthier C., Gaillard C., Quentin S., Maubert A.-L., Kogan S., De Thé H. (2019). FLT3-ITD Impedes Retinoic Acid, but Not Arsenic, Responses in Murine Acute Promyelocytic Leukemias. Blood.

[89] Lin R.J., Evans R.M. (2000). Acquisition of Oncogenic Potential by RAR Chimeras in Acute Promyelocytic Leukemia through Formation of Homodimers. Mol. Cell.

[90] Ablain J., de Thé H. (2014). Retinoic Acid Signaling in Cancer: The Parable of Acute Promyelocytic Leukemia: Retinoic Acid Signaling in Cancer. Int. J. Cancer.

[91] Zhu J., Nasr R., Pérès L., Riaucoux-Lormière F., Honoré N., Berthier C., Kamashev D., Zhou J., Vitoux D., Lavau C. (2007). RXR Is an Essential Component of the Oncogenic PML/RARA Complex In Vivo. Cancer Cell.

[92] Martens J.H.A., Brinkman A.B., Simmer F., Francoijs K.-J., Nebbioso A., Ferrara F., Altucci L., Stunnenberg H.G. (2010). PML-RARα/RXR Alters the Epigenetic Landscape in Acute Promyelocytic Leukemia. Cancer Cell.

[93] He L.-Z., Guidez F., Tribioli C., Peruzzi D., Ruthardt M., Zelent A., Pandolfi P.P. (1998). Distinct Interactions of PML-RARα and PLZF-RARα with Co-Repressors Determine Differential Responses to RA in APL. Nat. Genet..

[94] Kamashev D., Vitoux D., De Thé H. (2004). PML–RARA-RXR Oligomers Mediate Retinoid and Rexinoid/cAMP Cross-Talk in Acute Promyelocytic Leukemia Cell Differentiation. J. Exp. Med..

[95] Wang K., Wang P., Shi J., Zhu X., He M., Jia X., Yang X., Qiu F., Jin W., Qian M. (2010). PML/RARα Targets Promoter Regions Containing PU.1 Consensus and RARE Half Sites in Acute Promyelocytic Leukemia. Cancer Cell.

[96] Tan Y., Wang X., Song H., Zhang Y., Zhang R., Li S., Jin W., Chen S., Fang H., Chen Z. (2021). A PML/RARα Direct Target Atlas Redefines Transcriptional Deregulation in Acute Promyelocytic Leukemia. Blood.

[97] Villiers W., Kelly A., He X., Kaufman-Cook J., Elbasir A., Bensmail H., Lavender P., Dillon R., Mifsud B., Osborne C.S. (2023). Multi-Omics and Machine Learning Reveal Context-Specific Gene Regulatory Activities of PML::RARA in Acute Promyelocytic Leukemia. Nat. Commun..

[98] Geoffroy M.-C., de Thé H. (2020). Classic and Variants APLs, as Viewed from a Therapy Response. Cancers.

[99] Liquori A., Ibañez M., Sargas C., Sanz M., Barragán E., Cervera J. (2020). Acute Promyelocytic Leukemia: A Constellation of Molecular Events around a Single PML-RARA Fusion Gene. Cancers.

[100] Kastner P., Perez A., Lutz Y., Rochette-Egly C., Gaub M.P., Durand B., Lanotte M., Berger R., Chambon P. (1992). Structure, Localization and Transcriptional Properties of Two Classes of Retinoic Acid Receptor Alpha Fusion Proteins in Acute Promyelocytic Leukemia (APL): Structural Similarities with a New Family of Oncoproteins. EMBO J..

[101] Daniel M.T., Koken M., Romagné O., Barbey S., Bazarbachi A., Stadler M., Guillemin M.C., Degos L., Chomienne C., de Thé H. (1993). PML Protein Expression in Hematopoietic and Acute Promyelocytic Leukemia Cells. Blood.

[102] De Thé G., Riviere M., Bernhard W. (1960). Examen Au Microscope Électronique de La Tumeur VX2 Du Lapin Domestique Dérivée Du Papillome de Shope. Bull. Cancer.

[103] Fagioli M., Alcalay M., Pandolfi P.P., Venturini L., Mencarelli A., Simeone A., Acampora D., Grignani F., Pelicci P.G. (1992). Alternative Splicing of PML Transcripts Predicts Coexpression of Several Carboxy-Terminally Different Protein Isoforms. Oncogene.

[104] Koken M.H., Puvion-Dutilleul F., Guillemin M.C., Viron A., Linares-Cruz G., Stuurman N., De Jong L., Szostecki C., Calvo F., Chomienne C. (1994). The t(15;17) Translocation Alters a Nuclear Body in a Retinoic Acid-Reversible Fashion. EMBO J..

[105] Boisvert F.-M., Hendzel M.J., Bazett-Jones D.P. (2000). Promyelocytic Leukemia (Pml) Nuclear Bodies Are Protein Structures That Do Not Accumulate RNA. J. Cell Biol..

[106] Lallemand-Breitenbach V., De Thé H. (2010). PML Nuclear Bodies. Cold Spring Harb. Perspect. Biol..

[107] Bernardi R., Pandolfi P.P. (2007). Structure, Dynamics and Functions of Promyelocytic Leukaemia Nuclear Bodies. Nat. Rev. Mol. Cell Biol..

[108] Reymond A. (2001). The Tripartite Motif Family Identifies Cell Compartments. EMBO J..

[109] Meroni G., Desagher S. (2022). Cellular Function of TRIM E3 Ubiquitin Ligases in Health and Disease. Cells.

[110] Wang P., Benhenda S., Wu H., Lallemand-Breitenbach V., Zhen T., Jollivet F., Peres L., Li Y., Chen S.-J., Chen Z. (2018). RING Tetramerization Is Required for Nuclear Body Biogenesis and PML Sumoylation. Nat. Commun..

[111] Kamitani T., Kito K., Nguyen H.P., Wada H., Fukuda-Kamitani T., Yeh E.T.H. (1998). Identification of Three Major Sentrinization Sites in PML. J. Biol. Chem..

[112] Lallemand-Breitenbach V., Zhu J., Puvion F., Koken M., Honoré N., Doubeikovsky A., Duprez E., Pandolfi P.P., Puvion E., Freemont P. (2001). Role of Promyelocytic Leukemia (Pml) Sumolation in Nuclear Body Formation, 11s Proteasome Recruitment, and As_2_O_3_-Induced Pml or Pml/Retinoic Acid Receptor α Degradation. J. Exp. Med..

[113] Sahin U., de Thé H., Lallemand-Breitenbach V. (2014). PML Nuclear Bodies: Assembly and Oxidative Stress-Sensitive Sumoylation. Nucleus.

[114] Lallemand-Breitenbach V., Jeanne M., Benhenda S., Nasr R., Lei M., Peres L., Zhou J., Zhu J., Raught B., de Thé H. (2008). Arsenic Degrades PML or PML–RARα through a SUMO-Triggered RNF4/Ubiquitin-Mediated Pathway. Nat. Cell Biol..

[115] Van Damme E., Laukens K., Dang T.H., Van Ostade X. (2010). A Manually Curated Network of the PML Nuclear Body Interactome Reveals an Important Role for PML-NBs in SUMOylation Dynamics. Int. J. Biol. Sci..

[116] Sahin U., Ferhi O., Jeanne M., Benhenda S., Berthier C., Jollivet F., Niwa-Kawakita M., Faklaris O., Setterblad N., de Thé H. (2014). Oxidative Stress–Induced Assembly of PML Nuclear Bodies Controls Sumoylation of Partner Proteins. J. Cell Biol..

[117] Müller S., Matunis M.J., Dejean A. (1998). Conjugation with the Ubiquitin-Related Modifier SUMO-1 Regulates the Partitioning of PML within the Nucleus. EMBO J..

[118] Shen T.H., Lin H.-K., Scaglioni P.P., Yung T.M., Pandolfi P.P. (2006). The Mechanisms of PML-Nuclear Body Formation. Mol. Cell.

[119] Barroso-Gomila O., Trulsson F., Muratore V., Canosa I., Merino-Cacho L., Cortazar A.R., Pérez C., Azkargorta M., Iloro I., Carracedo A. (2021). Identification of Proximal SUMO-Dependent Interactors Using SUMO-ID. Nat. Commun..

[120] Hirano S., Udagawa O. (2022). SUMOylation Regulates the Number and Size of Promyelocytic Leukemia-Nuclear Bodies (PML-NBs) and Arsenic Perturbs SUMO Dynamics on PML by Insolubilizing PML in THP-1 Cells. Arch. Toxicol..

[121] Antolini F., Bello M.L., Sette M. (2003). Purified Promyelocytic Leukemia Coiled-Coil Aggregates as a Tetramer Displaying Low α-Helical Content. Protein Expr. Purif..

[122] Li Y., Ma X., Chen Z., Wu H., Wang P., Wu W., Cheng N., Zeng L., Zhang H., Cai X. (2019). B1 Oligomerization Regulates PML Nuclear Body Biogenesis and Leukemogenesis. Nat. Commun..

[123] Bercier P., Wang Q.Q., Zang N., Zhang J., Yang C., Maimaitiyiming Y., Abou-Ghali M., Berthier C., Wu C., Niwa-Kawakita M. (2023). Structural Basis of PML/RARA Oncoprotein Targeting by Arsenic Unravels a Cysteine Rheostat Controlling PML Body Assembly and Function. Cancer Discov..

[124] Guan D., Kao H.-Y. (2015). The Function, Regulation and Therapeutic Implications of the Tumor Suppressor Protein, PML. Cell Biosci..

[125] Weidtkamp-Peters S., Lenser T., Negorev D., Gerstner N., Hofmann T.G., Schwanitz G., Hoischen C., Maul G., Dittrich P., Hemmerich P. (2008). Dynamics of Component Exchange at PML Nuclear Bodies. J. Cell Sci..

[126] Tessier S., Ferhi O., Geoffroy M.-C., González-Prieto R., Canat A., Quentin S., Pla M., Niwa-Kawakita M., Bercier P., Rérolle D. (2022). Exploration of Nuclear Body-Enhanced Sumoylation Reveals That PML Represses 2-Cell Features of Embryonic Stem Cells. Nat. Commun..

[127] Cossec J.-C., Theurillat I., Chica C., Búa Aguín S., Gaume X., Andrieux A., Iturbide A., Jouvion G., Li H., Bossis G. (2018). SUMO Safeguards Somatic and Pluripotent Cell Identities by Enforcing Distinct Chromatin States. Cell Stem Cell.

[128] Hsu K.-S., Kao H.-Y. (2018). PML: Regulation and Multifaceted Function beyond Tumor Suppression. Cell Biosci..

[129] Lin H.-K., Bergmann S., Pandolfi P.P. (2004). Cytoplasmic PML Function in TGF-β Signalling. Nature.

[130] Wang Z.-G., Ruggero D., Ronchetti S., Zhong S., Gaboli M., Rivi R., Pandolfi P.P. (1998). Pml Is Essential for Multiple Apoptotic Pathways. Nat. Genet..

[131] Wang Z.G., Delva L., Gaboli M., Rivi R., Giorgio M., Cordon-Cardo C., Grosveld F., Pandolfi P.P. (1998). Role of PML in Cell Growth and the Retinoic Acid Pathway. Sci. New Ser..

[132] Koken M.H., Linares-Cruz G., Quignon F., Viron A., Chelbi-Alix M.K., Sobczak-Thépot J., Juhlin L., Degos L., Calvo F., de Thé H. (1995). The PML Growth-Suppressor Has an Altered Expression in Human Oncogenesis. Oncogene.

[133] Scaglioni P.P., Yung T.M., Cai L.F., Erdjument-Bromage H., Kaufman A.J., Singh B., Teruya-Feldstein J., Tempst P., Pandolfi P.P. (2006). A CK2-Dependent Mechanism for Degradation of the PML Tumor Suppressor. Cell.

[134] Trotman L.C., Alimonti A., Scaglioni P.P., Koutcher J.A., Cordon-Cardo C., Pandolfi P.P. (2006). Identification of a Tumour Suppressor Network Opposing Nuclear Akt Function. Nature.

[135] Wolyniec K., Shortt J., de Stanchina E., Levav-Cohen Y., Alsheich-Bartok O., Louria-Hayon I., Corneille V., Kumar B., Woods S.J., Opat S. (2012). E6AP Ubiquitin Ligase Regulates PML-Induced Senescence in Myc-Driven Lymphomagenesis. Blood.

[136] Haupt S., Mitchell C., Corneille V., Shortt J., Fox S., Pandolfi P.P., Castillo-Martin M., Bonal D., Cordon-Carlo C., Lozano G. (2013). Loss of PML Cooperates with Mutant P53 to Drive More Aggressive Cancers in a Gender-Dependent Manner. Cell Cycle.

[137] Zhang P., Chin W., Chow L.T., Chan A.S., Yim A.P., Leung S.F., Mok T.S., Chang K.S., Johnson P.J., Chan J.Y. (2000). Lack of Expression for the Suppressor PML in Human Small Cell Lung Carcinoma. Int. J. Cancer.

[138] Gurrieri C., Capodieci P., Bernardi R., Scaglioni P.P., Nafa K., Rush L.J., Verbel D.A., Cordon-Cardo C., Pandolfi P.P. (2004). Loss of the Tumor Suppressor PML in Human Cancers of Multiple Histologic Origins. JNCI J. Natl. Cancer Inst..

[139] Datta N., Islam S., Chatterjee U., Chatterjee S., Panda C.K., Ghosh M.K. (2019). Promyelocytic Leukemia (PML) Gene Regulation: Implication towards Curbing Oncogenesis. Cell Death Dis..

[140] Rego E.M., Wang Z.G., Peruzzi D., He L.Z., Cordon-Cardo C., Pandolfi P.P. (2001). Role of Promyelocytic Leukemia (PML) Protein in Tumor Suppression. J. Exp. Med..

[141] Voisset E., Moravcsik E., Stratford E.W., Jaye A., Palgrave C.J., Hills R.K., Salomoni P., Kogan S.C., Solomon E., Grimwade D. (2018). Pml Nuclear Body Disruption Cooperates in APL Pathogenesis and Impairs DNA Damage Repair Pathways in Mice. Blood.

[142] Ferbeyre G., de Stanchina E., Querido E., Baptiste N., Prives C., Lowe S.W. (2000). PML Is Induced by Oncogenic Ras and Promotes Premature Senescence. Genes Dev..

[143] Pearson M., Carbone R., Sebastiani C., Cioce M., Fagioli M., Saito S., Higashimoto Y., Appella E., Minucci S., Pandolfi P.P. (2000). PML Regulates P53 Acetylation and Premature Senescence Induced by Oncogenic Ras. Nature.

[144] Bischof O. (2002). Deconstructing PML-Induced Premature Senescence. EMBO J..

[145] Bernardi R., Papa A., Pandolfi P.P. (2008). Regulation of Apoptosis by PML and the PML-NBs. Oncogene.

[146] Vernier M., Bourdeau V., Gaumont-Leclerc M.-F., Moiseeva O., Begin V., Saad F., Mes-Masson A.-M., Ferbeyre G. (2011). Regulation of E2Fs and Senescence by PML Nuclear Bodies. Genes Dev..

[147] Talluri S., Dick F.A. (2014). The Retinoblastoma Protein and PML Collaborate to Organize Heterochromatin and Silence E2F-Responsive Genes during Senescence. Cell Cycle.

[148] Ablain J., Rice K., Soilihi H., de Reynies A., Minucci S., de Thé H. (2014). Activation of a Promyelocytic Leukemia–Tumor Protein 53 Axis Underlies Acute Promyelocytic Leukemia Cure. Nat. Med..

[149] de Thé H., Pandolfi P.P., Chen Z. (2017). Acute Promyelocytic Leukemia: A Paradigm for Oncoprotein-Targeted Cure. Cancer Cell.

[150] Liebl M.C., Hofmann T.G. (2022). Regulating the P53 Tumor Suppressor Network at PML Biomolecular Condensates. Cancers.

[151] Ivanschitz L., Takahashi Y., Jollivet F., Ayrault O., Le Bras M., de Thé H. (2015). PML IV/ARF Interaction Enhances P53 SUMO-1 Conjugation, Activation, and Senescence. Proc. Natl. Acad. Sci. USA.

[152] Dellaire G. (2006). The Number of PML Nuclear Bodies Increases in Early S Phase by a Fission Mechanism. J. Cell Sci..

[153] Corpet A., Kleijwegt C., Roubille S., Juillard F., Jacquet K., Texier P., Lomonte P. (2020). PML Nuclear Bodies and Chromatin Dynamics: Catch Me If You Can!. Nucleic Acids Res..

[154] Delbarre E., Ivanauskiene K., Spirkoski J., Shah A., Vekterud K., Moskaug J.Ø., Bøe S.O., Wong L.H., Küntziger T., Collas P. (2017). PML Protein Organizes Heterochromatin Domains Where It Regulates Histone H3.3 Deposition by ATRX/DAXX. Genome Res..

[155] Fracassi C., Ugge’ M., Abdelhalim M., Zapparoli E., Simoni M., Magliulo D., Mazza D., Lazarevic D., Morelli M.J., Collas P. (2023). PML Modulates Epigenetic Composition of Chromatin to Regulate Expression of Pro-Metastatic Genes in Triple-Negative Breast Cancer. Nucleic Acids Res..

[156] Ishov A.M., Sotnikov A.G., Negorev D., Vladimirova O.V., Neff N., Kamitani T., Yeh E.T.H., Strauss J.F., Maul G.G. (1999). Pml Is Critical for Nd10 Formation and Recruits the Pml-Interacting Protein Daxx to This Nuclear Structure When Modified by Sumo-1. J. Cell Biol..

[157] Boisvert F.-M., Kruhlak M.J., Box A.K., Hendzel M.J., Bazett-Jones D.P. (2001). The Transcription Coactivator Cbp Is a Dynamic Component of the Promyelocytic Leukemia Nuclear Body. J. Cell Biol..

[158] D’Orazi G., Cecchinelli B., Bruno T., Manni I., Higashimoto Y., Saito S., Gostissa M., Coen S., Marchetti A., Del Sal G. (2002). Homeodomain-Interacting Protein Kinase-2 Phosphorylates P53 at Ser 46 and Mediates Apoptosis. Nat. Cell Biol..

[159] Hofmann T.G., Möller A., Sirma H., Zentgraf H., Taya Y., Dröge W., Will H., Schmitz M.L. (2002). Regulation of P53 Activity by Its Interaction with Homeodomain-Interacting Protein Kinase-2. Nat. Cell Biol..

[160] Gao Q., Liang W.-W., Foltz S.M., Mutharasu G., Jayasinghe R.G., Cao S., Liao W.-W., Reynolds S.M., Wyczalkowski M.A., Yao L. (2018). Driver Fusions and Their Implications in the Development and Treatment of Human Cancers. Cell Rep..

[161] Tripathi S., Shirnekhi H.K., Gorman S.D., Chandra B., Baggett D.W., Park C.-G., Somjee R., Lang B., Hosseini S.M.H., Pioso B.J. (2023). Defining the Condensate Landscape of Fusion Oncoproteins. Nat. Commun..

[162] Cambiaghi V., Giuliani V., Lombardi S., Marinelli C., Toffalorio F., Pelicci P.G., Meroni G. (2012). TRIM Proteins in Cancer. TRIM/RBCC Proteins.

[163] Crawford L.J., Johnston C.K., Irvine A.E. (2018). TRIM Proteins in Blood Cancers. J. Cell Commun. Signal..

[164] Occhionorelli M., Santoro F., Pallavicini I., Gruszka A., Moretti S., Bossi D., Viale A., Shing D., Ronzoni S., Muradore I. (2011). The Self-Association Coiled-Coil Domain of PML Is Sufficient for the Oncogenic Conversion of the Retinoic Acid Receptor (RAR) Alpha. Leukemia.

[165] Li Y., Ma X., Wu W., Chen Z., Meng G. (2020). PML Nuclear Body Biogenesis, Carcinogenesis, and Targeted Therapy. Trends Cancer.

[166] Kwok C., Zeisig B.B., Dong S., So C.W.E. (2006). Forced Homo-Oligomerization of RARα Leads to Transformation of Primary Hematopoietic Cells. Cancer Cell.

[167] Sternsdorf T., Phan V.T., Maunakea M.L., Ocampo C.B., Sohal J., Silletto A., Galimi F., Le Beau M.M., Evans R.M., Kogan S.C. (2006). Forced Retinoic Acid Receptor α Homodimers Prime Mice for APL-like Leukemia. Cancer Cell.

[168] Zhu J., Zhou J., Peres L., Riaucoux F., Honoré N., Kogan S., de Thé H. (2005). A Sumoylation Site in PML/RARA Is Essential for Leukemic Transformation. Cancer Cell.

[169] Vitaliano-Prunier A., Halftermeyer J., Ablain J., de Reynies A., Peres L., Le Bras M., Metzger D., de The H. (2014). Clearance of PML/RARA-Bound Promoters Suffice to Initiate APL Differentiation. Blood.

[170] Grignani F., De Matteis S., Nervi C., Tomassoni L., Gelmetti V., Cioce M., Fanelli M., Ruthardt M., Ferrara F.F., Zamir I. (1998). Fusion Proteins of the Retinoic Acid Receptor-α Recruit Histone Deacetylase in Promyelocytic Leukaemia. Nature.

[171] Tiefenbach J., Novac N., Ducasse M., Eck M., Melchior F., Heinzel T. (2006). SUMOylation of the Corepressor N-CoR Modulates Its Capacity to Repress Transcription. Mol. Biol. Cell.

[172] Lin R.J., Nagy L., Inoue S., Shao W., Miller W.H., Evans R.M. (1998). Role of the Histone Deacetylase Complex in Acute Promyelocytic Leukaemia. Nature.

[173] Wang P., Tang Z., Lee B., Zhu J.J., Cai L., Szalaj P., Tian S.Z., Zheng M., Plewczynski D., Ruan X. (2020). Chromatin Topology Reorganization and Transcription Repression by PML-RARα in Acute Promyeloid Leukemia. Genome Biol..

[174] Iaccarino L., Ottone T., Divona M., Cicconi L., Cairoli R., Voso M.T., Lo-Coco F. (2016). Mutations Affecting Both the Rearranged and the Unrearranged *PML* Alleles in Refractory Acute Promyelocytic Leukaemia. Br. J. Haematol..

[175] Hadjimichael C., Chanoumidou K., Nikolaou C., Klonizakis A., Theodosi G.-I., Makatounakis T., Papamatheakis J., Kretsovali A. (2017). Promyelocytic Leukemia Protein Is an Essential Regulator of Stem Cell Pluripotency and Somatic Cell Reprogramming. Stem Cell Rep..

[176] Amodeo V., Deli A., Betts J., Bartesaghi S., Zhang Y., Richard-Londt A., Ellis M., Roshani R., Vouri M., Galavotti S. (2017). A PML/Slit Axis Controls Physiological Cell Migration and Cancer Invasion in the CNS. Cell Rep..

[177] Lapidot T., Sirard C., Vormoor J., Murdoch B., Hoang T., Caceres-Cortes J., Minden M., Paterson B., Caligiuri M.A., Dick J.E. (1994). A Cell Initiating Human Acute Myeloid Leukaemia after Transplantation into SCID Mice. Nature.

[178] Bonnet D., Dick J.E. (1997). Human Acute Myeloid Leukemia Is Organized as a Hierarchy That Originates from a Primitive Hematopoietic Cell. Nat. Med..

[179] Shlush L.I., Mitchell A., Heisler L., Abelson S., Ng S.W.K., Trotman-Grant A., Medeiros J.J.F., Rao-Bhatia A., Jaciw-Zurakowsky I., Marke R. (2017). Tracing the Origins of Relapse in Acute Myeloid Leukaemia to Stem Cells. Nature.

[180] O’Reilly E., Zeinabad H.A., Szegezdi E. (2021). Hematopoietic versus Leukemic Stem Cell Quiescence: Challenges and Therapeutic Opportunities. Blood Rev..

[181] Ito K., Bernardi R., Morotti A., Matsuoka S., Saglio G., Ikeda Y., Rosenblatt J., Avigan D.E., Teruya-Feldstein J., Pandolfi P.P. (2008). PML Targeting Eradicates Quiescent Leukaemia-Initiating Cells. Nature.

[182] Ito K., Carracedo A., Weiss D., Arai F., Ala U., Avigan D.E., Schafer Z.T., Evans R.M., Suda T., Lee C.-H. (2012). A PML–PPAR-δ Pathway for Fatty Acid Oxidation Regulates Hematopoietic Stem Cell Maintenance. Nat. Med..

[183] Carracedo A., Weiss D., Leliaert A.K., Bhasin M., de Boer V.C.J., Laurent G., Adams A.C., Sundvall M., Song S.J., Ito K. (2012). A Metabolic Prosurvival Role for PML in Breast Cancer. J. Clin. Investig..

[184] Martín-Martín N., Piva M., Urosevic J., Aldaz P., Sutherland J.D., Fernández-Ruiz S., Arreal L., Torrano V., Cortazar A.R., Planet E. (2016). Stratification and Therapeutic Potential of PML in Metastatic Breast Cancer. Nat. Commun..

[185] Gentric G., Kieffer Y., Mieulet V., Goundiam O., Bonneau C., Nemati F., Hurbain I., Raposo G., Popova T., Stern M.-H. (2019). PML-Regulated Mitochondrial Metabolism Enhances Chemosensitivity in Human Ovarian Cancers. Cell Metab..

[186] Arreal L., Piva M., Fernández S., Revandkar A., Schaub- Clerigué A., Villanueva J., Zabala-Letona A., Pujana M., Astobiza I., Cortazar A.R. (2020). Targeting PML in Triple Negative Breast Cancer Elicits Growth Suppression and Senescence. Cell Death Differ..

[187] Zhu J., Koken M.H.M., Quignon F., Chelbi-Alix M.K., Degos L., Wang Z.Y., Chen Z., de The H. (1997). Arsenic-Induced PML Targeting onto Nuclear Bodies: Implications for the Treatment of Acute Promyelocytic Leukemia. Proc. Natl. Acad. Sci. USA.

[188] Islam K., Wang Q.Q., Naranmandura H. (2015). Molecular Mechanisms of Arsenic Toxicity. Advances in Molecular Toxicology.

[189] Jeanne M., Lallemand-Breitenbach V., Ferhi O., Koken M., Le Bras M., Duffort S., Peres L., Berthier C., Soilihi H., Raught B. (2010). PML/RARA Oxidation and Arsenic Binding Initiate the Antileukemia Response of As_2_O_3_. Cancer Cell.

[190] Niwa-Kawakita M., Ferhi O., Soilihi H., Le Bras M., Lallemand-Breitenbach V., de Thé H. (2017). PML Is a ROS Sensor Activating P53 upon Oxidative Stress. J. Exp. Med..

[191] Tatham M.H., Geoffroy M.-C., Shen L., Plechanovova A., Hattersley N., Jaffray E.G., Palvimo J.J., Hay R.T. (2008). RNF4 Is a Poly-SUMO-Specific E3 Ubiquitin Ligase Required for Arsenic-Induced PML Degradation. Nat. Cell Biol..

[192] Erker Y., Neyret-Kahn H., Seeler J.S., Dejean A., Atfi A., Levy L. (2013). Arkadia, a Novel SUMO-Targeted Ubiquitin Ligase Involved in PML Degradation. Mol. Cell. Biol..

[193] Tsai J.M., Aguirre J.D., Li Y.-D., Brown J., Focht V., Kater L., Kempf G., Sandoval B., Schmitt S., Rutter J.C. (2023). UBR5 Forms Ligand-Dependent Complexes on Chromatin to Regulate Nuclear Hormone Receptor Stability. Mol. Cell.

[194] Jaffray E.G., Tatham M.H., Mojsa B., Liczmanska M., Rojas-Fernandez A., Yin Y., Ball G., Hay R.T. (2023). The P97/VCP Segregase Is Essential for Arsenic-Induced Degradation of PML and PML-RARA. J. Cell Biol..

[195] de Thé H. (2018). Differentiation Therapy Revisited. Nat. Rev. Cancer.

[196] Yilmaz M., Kantarjian H., Ravandi F. (2021). Acute Promyelocytic Leukemia Current Treatment Algorithms. Blood Cancer J..

[197] Gallagher R.E., Moser B.K., Racevskis J., Poiré X., Bloomfield C.D., Carroll A.J., Ketterling R.P., Roulston D., Schachter-Tokarz E., Zhou D.-C. (2012). Treatment-Influenced Associations of PML-RARα Mutations, FLT3 Mutations, and Additional Chromosome Abnormalities in Relapsed Acute Promyelocytic Leukemia. Blood.

[198] Raelson J.V., Nervi C., Rosenauer A., Benedetti L., Monczak Y., Pearson M., Pelicci P.G., Miller W.H. (1996). The PML/RAR Alpha Oncoprotein Is a Direct Molecular Target of Retinoic Acid in Acute Promyelocytic Leukemia Cells. Blood.

[199] Zhu J., Gianni M., Kopf E., Honore N., Chelbi-Alix M., Koken M., Quignon F., Rochette-Egly C., de The H. (1999). Retinoic Acid Induces Proteasome-Dependent Degradation of Retinoic Acid Receptor Alpha (RARalpha) and Oncogenic RARalpha Fusion Proteins. Proc. Natl. Acad. Sci. USA.

[200] Ablain J., Leiva M., Peres L., Fonsart J., Anthony E., de Thé H. (2013). Uncoupling RARA Transcriptional Activation and Degradation Clarifies the Bases for APL Response to Therapies. J. Exp. Med..

[201] Nasr R., Guillemin M.-C., Ferhi O., Soilihi H., Peres L., Berthier C., Rousselot P., Robledo-Sarmiento M., Lallemand-Breitenbach V., Gourmel B. (2008). Eradication of Acute Promyelocytic Leukemia-Initiating Cells through PML-RARA Degradation. Nat. Med..

[202] McKenzie M.D., Ghisi M., Oxley E.P., Ngo S., Cimmino L., Esnault C., Liu R., Salmon J.M., Bell C.C., Ahmed N. (2019). Interconversion between Tumorigenic and Differentiated States in Acute Myeloid Leukemia. Cell Stem Cell.

[203] Douer D., Estey E., Santillana S., Bennett J.M., Lopez-Bernstein G., Boehm K., Williams T. (2001). Treatment of Newly Diagnosed and Relapsed Acute Promyelocytic Leukemia with Intravenous Liposomal All-Transretinoic Acid. Blood.

[204] Tsimberidou A.-M., Tirado-Gomez M., Andreeff M., O’Brien S., Kantarjian H., Keating M., Lopez-Berestein G., Estey E. (2006). Single-Agent Liposomal All-Trans Retinoic Acid Can Cure Some Patients with Untreated Acute Promyelocytic Leukemia: An Update of The University of Texas M. D. Anderson Cancer Center Series. Leuk. Lymphoma.

[205] Rabellino A., Scaglioni P.P. (2013). PML Degradation: Multiple Ways to Eliminate PML. Front. Oncol..

[206] Kchour G., Tarhini M., Kooshyar M.-M., El Hajj H., Wattel E., Mahmoudi M., Hatoum H., Rahimi H., Maleki M., Rafatpanah H. (2009). Phase 2 Study of the Efficacy and Safety of the Combination of Arsenic Trioxide, Interferon Alpha, and Zidovudine in Newly Diagnosed Chronic Adult T-Cell Leukemia/Lymphoma (ATL). Blood.

[207] Dassouki Z., Sahin U., El Hajj H., Jollivet F., Kfoury Y., Lallemand-Breitenbach V., Hermine O., Bazarbachi A. (2015). ATL Response to Arsenic/Interferon Therapy Is Triggered by SUMO/PML/RNF4-Dependent Tax Degradation. Blood.

[208] Marçais A., Cook L., Witkover A., Asnafi V., Avettand-Fenoel V., Delarue R., Cheminant M., Sibon D., Frenzel L., de Thé H. (2020). Arsenic Trioxide (As_2_O_3_) as a Maintenance Therapy for Adult T Cell Leukemia/Lymphoma. Retrovirology.

[209] Hleihel R., El Hajj H., Wu H.-C., Berthier C., Zhu H.-H., Massoud R., Chakhachiro Z., El Sabban M., De The H., Bazarbachi A. (2021). A Pin1/PML/P53 Axis Activated by Retinoic Acid in *NPM-1c* Acute Myeloid Leukemia. Haematologica.

[210] Schlenk R.F., Dohner K., Kneba M., Gotze K., Hartmann F., del Valle F., Kirchen H., Koller E., Fischer J.T., Bullinger L. (2009). Gene Mutations and Response to Treatment with All-Trans Retinoic Acid in Elderly Patients with Acute Myeloid Leukemia. Results from the AMLSG Trial AML HD98B. Haematologica.

[211] Wu H.-C., Rérolle D., Berthier C., Hleihel R., Sakamoto T., Quentin S., Benhenda S., Morganti C., Wu C., Conte L. (2021). Actinomycin D Targets NPM1c-Primed Mitochondria to Restore PML-Driven Senescence in AML Therapy. Cancer Discov..

[212] Okazaki T., Kageji T., Kuwayama K., Kitazato K.T., Mure H., Hara K., Morigaki R., Mizobuchi Y., Matsuzaki K., Nagahiro S. (2012). Up-Regulation of Endogenous PML Induced by a Combination of Interferon-Beta and Temozolomide Enhances P73/YAP-Mediated Apoptosis in Glioblastoma. Cancer Lett..

[213] Dagher T., Maslah N., Edmond V., Cassinat B., Vainchenker W., Giraudier S., Pasquier F., Verger E., Niwa-Kawakita M., Lallemand-Breitenbach V. (2021). JAK2V617F Myeloproliferative Neoplasm Eradication by a Novel Interferon/Arsenic Therapy Involves PML. J. Exp. Med..

[214] Zhou W., Cheng L., Shi Y., Ke S.Q., Huang Z., Fang X., Chu C., Xie Q., Bian X., Rich J.N. (2015). Arsenic Trioxide Disrupts Glioma Stem Cells via Promoting PML Degradation to Inhibit Tumor Growth. Oncotarget.

[215] Voon H.P.J., Hii L., Garvie A., Udugama M., Krug B., Russo C., Chüeh A.C., Daly R.J., Morey A., Bell T.D.M. (2023). Pediatric Glioma Histone H3.3 K27M/G34R Mutations Drive Abnormalities in PML Nuclear Bodies. Genome Biol..

[216] Khetchoumian K., Teletin M., Tisserand J., Mark M., Herquel B., Ignat M., Zucman-Rossi J., Cammas F., Lerouge T., Thibault C. (2007). Loss of Trim24 (Tif1α) Gene Function Confers Oncogenic Activity to Retinoic Acid Receptor Alpha. Nat. Genet..

[217] Tan J., Ong C.K., Lim W.K., Ng C.C.Y., Thike A.A., Ng L.M., Rajasegaran V., Myint S.S., Nagarajan S., Thangaraju S. (2015). Genomic Landscapes of Breast Fibroepithelial Tumors. Nat. Genet..

[218] McKeown M.R., Corces M.R., Eaton M.L., Fiore C., Lee E., Lopez J.T., Chen M.W., Smith D., Chan S.M., Koenig J.L. (2017). Superenhancer Analysis Defines Novel Epigenomic Subtypes of Non-APL AML, Including an RARα Dependency Targetable by SY-1425, a Potent and Selective RARα Agonist. Cancer Discov..

[219] Lübbert M., Grishina O., Schmoor C., Schlenk R.F., Jost E., Crysandt M., Heuser M., Thol F., Salih H.R., Schittenhelm M.M. (2020). Valproate and Retinoic Acid in Combination With Decitabine in Elderly Nonfit Patients With Acute Myeloid Leukemia: Results of a Multicenter, Randomized, 2 × 2, Phase II Trial. J. Clin. Oncol..

[220] De Botton S., Cluzeau T., Vigil C., Cook R.J., Rousselot P., Rizzieri D.A., Liesveld J.L., Fenaux P., Braun T., Banos A. (2023). Targeting *RARA* Overexpression with Tamibarotene, a Potent and Selective RARα Agonist, Is a Novel Approach in AML. Blood Adv..

[221] Zhao J., Liang J.-W., Xue H.-L., Shen S.-H., Chen J., Tang Y.-J., Yu L.-S., Liang H.-H., Gu L.-J., Tang J.-Y. (2019). The Genetics and Clinical Characteristics of Children Morphologically Diagnosed as Acute Promyelocytic Leukemia. Leukemia.

